# A Multitask Learning Model with Multiperspective Attention and Its Application in Recommendation

**DOI:** 10.1155/2021/8550270

**Published:** 2021-10-15

**Authors:** Yingshuai Wang, Dezheng Zhang, Aziguli Wulamu

**Affiliations:** ^1^Department of Computer, School of Computer and Communication Engineering, University of Science and Technology Beijing (USTB), Beijing 100083, China; ^2^Beijing Key Laboratory of Knowledge Engineering for Materials Science Beijing, University of Science and Technology Beijing (USTB), Beijing 100083, China

## Abstract

Training models to predict click and order targets at the same time. For better user satisfaction and business effectiveness, multitask learning is one of the most important methods in e-commerce. Some existing researches model user representation based on historical behaviour sequence to capture user interests. It is often the case that user interests may change from their past routines. However, multi-perspective attention has broad horizon, which covers different characteristics of human reasoning, emotions, perception, attention, and memory. In this paper, we attempt to introduce the multi-perspective attention and sequence behaviour into multitask learning. Our proposed method offers better understanding of user interest and decision. To achieve more flexible parameter sharing and maintaining the special feature advantage of each task, we improve the attention mechanism at the view of expert interactive. To the best of our knowledge, we firstly propose the implicit interaction mode, the explicit hard interaction mode, the explicit soft interaction mode, and the data fusion mode in multitask learning. We do experiments on public data and lab medical data. The results show that our model consistently achieves remarkable improvements to the state-of-the-art method.

## 1. Introduction

In the real world, there are some scenarios for multitask learning. In the e-commerce field, we need to increase click through rate (CTR) and order conversion rate (CVR) at the same time. In the music field, we need to improve the song opening rate and the effective playback rate. In the Chinese medical case recommendation, we need to improve the click rate of medical records and the user satisfaction. To improve the recommendation accuracy, Chen et al. [1] propose an improved collaborative filtering algorithm, which introduces the Bhattacharyya similarity calculation into the traditional calculation formula. However, the single-task learning cannot take into account multiple indicators at the same time. In this context, the study of multitask learning emerges. On the bases of shared bottom, multi-gate mixture of experts (MMOE) [2] designs different gate networks for different tasks. By updating the weights of experts, it is better to describe the characteristics about all tasks. It has an improved effect on some tasks that are not very related to each other. In video recommendation, in order to improve user engagement and user satisfaction, Zhao et al. [3] propose shallow subnetwork. It also solves the online and offline problem of sample bias. As is known to all, order behaviour occurs after the click action. The model training process is performed in the click sample subspace, and applied in the entire space online, which will cause sample deviation. Wen et al. [4] add these intermediate behaviours to the model by improving loss function. Previous multitask learning manually turns hyperparameters, which could not balance the network flexibility and performance cost. Subnetwork routing (SNR) [5] is not sensitive to the strength of the correlation between tasks. It can be combined to learn a good structure and can realize flexible parameter sharing. Qin et al. [6] propose a model, which can combine MMOE and Long Short-Term Memory (LSTM) together. The model applies user behaviour sequence feature in multitask learning scenarios. Real application scenarios always face the challenge of data sparsity, data heterogeneity, and complex multiobjective, which the MMOE and LSTM try to solve. The Progressive Layered Extraction (PLE) [7] network is proposed, whose purpose is to leverage the seesaw phenomenon in multitask learning. To solve the negative transformer problem, on the one hand, PLE model splits the experts into shared expert and private expert; on the other hand, PLE model divides the sample space by loss function. Wang et al. [8] propose a Multitask-Aware Fairness (MTAF) method to improve fairness in multitask learning. Xi et al. [9] propose an Adaptive Information Transfer Multitask (AITM) framework, which constructs the sequential dependence among multistep conversions by the Adaptive Information Transfer (AIT) module. Low-rank decomposed self-attention network (Light-SAN) [10] is proposed, which learns the context-aware representation via users' history items and mines sequential relations among items efficiently. Gating-Enhanced Multitask Neural Networks (Gem-NN) [11] design a gating mechanism between embedding layer and MLP, which learns feature interaction and manages information flow. Multiple-Level Sparse Sharing Model (MSSM) [12] is proposed, which includes a field-level sparse connection module (FSCM) and a cell-level sparse sharing module (CSSM). The FSCM can learn features selectively and the CSSM can share knowledge across all tasks efficiently. To resolve the selection bias and data sparsity issue, Hierarchically Modelling both Micro and Macro behaviour (HM^3^) [13] is proposed for CVR prediction, which employs micro and macro post-click behaviour in a multitask learning mode. Zhao et al. [14] propose multiple relational attention network, which employs attention mechanism to improve prediction accuracy. The model structure comes from three perspectives: the first is task and feature, the second is feature and feature, and the third is task and task. In recommendation systems, the pareto algorithm is applied to the multiobjective learning, which can make at least one objective better without harming the other objective. The loss function refers to the KKT condition and the relax constraints, and then the model updates the weights at each batch. With the idea of knowledge distillation, Tang et al. [15] propose a novel model, which employs dominant feature to guide multitask learning. The feature matching algorithm combines original feature and dominant feature, which maps them to a new hidden space and improves the efficiency of multitask information sharing. Wang et al. [16] propose a new model to improve relation extraction algorithm. The embedding layer represents sharing information, which uses Bidirectional Encoder Representation from Transformer (BERT) pretrained model as initial computing part. The model introduces knowledge distillation to use the information of auxiliary tasks better. According to the multitask learning framework, Shao et al. [17] introduce attention map convolutional layer to mine the bilateral high-order feature graph from user and commodity. The model can dynamically capture the users' implicit interest for commodity. Yao et al. [18] propose a strong aggregation multitask learning method, which can group tasks by learning representation vectors. This method assumes that one task is a linear combination of other tasks. The correlation between tasks is calculated through the statistical coefficient. Based on the knowledge graph, Yu et al. [19] propose a multitask feature learning method using the knowledge graph to calculate the embedding vector assist the recommendation task finally. Conversation recommendation is becoming an important part of e-commerce. In order to improve the prediction effect via mining sequence feature, Chen et al. [20] employ the graph structure cascade and node sequence diffusion. The model proposes a sharing representation layer, which helps to understand the task of cascading relationship. The sequence knowledge is learned from the share representation layer, which can encode the cascade structure and sequence node well. Most multitask build network through multilayer feature sharing.

However, the above studies in multitask learning are based on feature engineering and knowledge representation, without introducing multi-perspective attention. We integrate coarse-grained attention, fine-grained attention, boosting expert mode, and expert-level self-attention; therefore, different task experts can interact better.

The rest of this paper is organized as follows.  Section 2 introduces application of recommendation system in academic and industry.  Section 3 discusses the recall stage, the ranking stage, and the diversity stage in the recommender system, and describes our specific improvement methods.  Section 4 makes experiment in public data set, and compares the baseline.  Section 5 draws conclusion and proposes prospects.

The main contributions of our proposed model are summarized as follows:

1. We introduce coarse-grained attention and fine-grained attention in the gate network. Each task layer learns a query vector for each expert, and inner product is taken on the query vector and the expert, then regarding the result as the attention. The gate attention methods achieve better performance than the base MMOE.
2. Inspired by the fact that the gradient boosting tree is better than random forest, we design the gradient boosting expert network, which enhances the interaction among different experts.
3. To the best of our knowledge, we are the first to introduce the expert-level multi-head self-attention into multitask learning and get better effectiveness.
4. We design the time-space sequence feature into multitask learning and improve the loss function, which can support multiple-source datasets.
5. We conduct extensive experiments on Ali-CCP data and confirm the superiority of our proposed model over representative state-of-the-art method.

## 2. Related Work

### 2.1. Multitask Learning Architecture

In the deep neural network, the click task and the order task are weighted in different proportions, and then they are processed as positive samples. The idea of a single-task model is difficult to find trade-off between click and order tasks. The model pays more attention to a certain part so that it learns information perhaps deviated from the original sample distribution. In addition, the single-task processing ignores some information, which contains rich correlation among tasks. Use multitask learning to optimize multiple targets at the same time. Share parameters to learn correlation. Subtask learns the differences of the sample distribution. By this way, we improve generation ability of the model.

**Figure 1:**
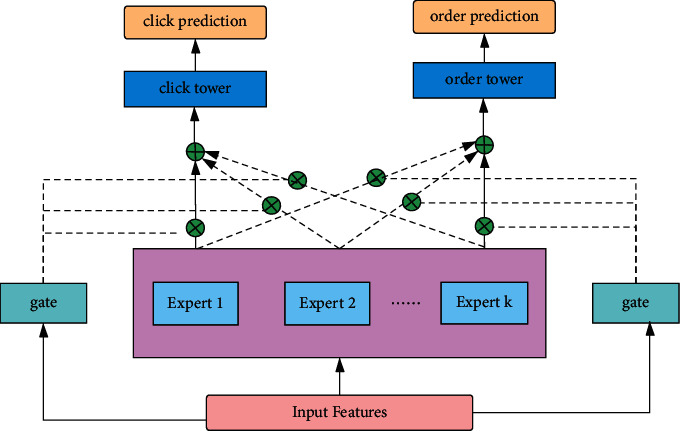
The multi-gate multi-expert network.

As is known to all, most multitask learning networks have feature parameter sharing module, which is divided into hard sharing and soft sharing specifically. Hard sharing feature is constructed at the bottom layer and completely shared. The upper layer introduces different networks so as to predict their respective tasks. When the tasks are more relevant, hard sharing is much more effective. Negative transfer will occur when tasks are not very relevant. If the effect of one task increases, the effect of another task decreases. In order to solve this problem, Google proposes MMOE model. The model constructs gate control mechanism for each task, which brings better effects. Tencent proposes the PLE model. Trying to introduce multiple layers of shared experts and private experts resolves the heterogeneous relationship between tasks furtherly. The structure of MMOE model is shown in Figure 1.

(1) $$\begin{array}{c} y_{k}=h^{k}\left(f^{k}\left(x\right)\right),\\ f^{k}\left(x\right)=\sum_{i=1}^{n}g^{k}{\left(x\right)}_{i}f_{i}\left(x\right), \end{array}$$

where ∑_*i*=1_^*n*^*g*^*k*^(*x*)_*i*_=1, *g*^*k*^(*x*)_*i*_ represents the output logits of gating at the *i*th expert, which is used to calculate the weight of the *i*th expert. *f*_*i*_(*x*) denotes the *i*th expert network; *h*^*k*^(.) means the hidden layer. Furtherly, the gate network equation is as follows:

(2) $$\begin{array}{c} g^{k}\left(x\right)=\text{softmax}\left(W_{gk}x\right). \end{array}$$

#### 2.1.1. Expert Network Part

*Step 1*. Construct a neural network for each expert and get the output *y*.
  (3) $$\begin{array}{c} y=X*{\text{hidden}}_{1}*{\text{hidden}}_{2}, \end{array}$$
where *X* means the input features, whose shape is [batch size, feature size]. hidden_1_ indicates the units of the first expert hidden layer, with the shape of [feature size, units of the first hidden layer]. hidden_2_ shows the units of the second expert hidden layer, with the shape of [units of the first hidden layer, units of the second hidden layer]. As a result, the shape of *y* is [batch size, units of the second hidden layer].
*Step 2*. Build a list of expert outputs, which is used to restore the output of each expert.
*Step 3*. In the last dimension of expert output, we use flatten operation to stack the *y*; then we store it as a tensor. The tensor shape is [batch size, units of the second hidden layer, the number of experts].

#### 2.1.2. Gate Network Part

*Step 1*. Construct a neural network for each gate and get the gate output *y*.
  (4) $$\begin{array}{c} y=X*{\text{hidden}}_{1}*{\text{hidden}}_{2}, \end{array}$$
where *X* means the input features, whose shape is [batch size, feature size]. hidden_1_ indicates the units of the first gate hidden layer, with the shape of [feature size, units of the first hidden layer]. hidden_2_ shows the units of the second gate hidden layer, with the shape of [units of the first gate hidden layer, units of the second gate hidden layer]. As a result, the shape of *y* is [batch size, units of the second gate hidden layer].
*Step 2*. Construct a gate dictionary named gates output, whose key is the task name and whose value is the output *y* of the last gate network layer.
  (5) $$\begin{array}{c} \text{gates}\_\text{output}\left[\text{task}\right]=y. \end{array}$$
*Step 3*. Convert gate output into weights, *y* is expanded on the axis index-1. After that, the number of neurons in the last layer of expert is copied as weights matrix. The shape of weights matrix is [batch size, units of the second expert hidden layer, units of the second gate hidden layer].
*Step 4*. Using expert output and gating weights, we calculate the tensor which is connected to the tower. Both the expert output after stacking and the weights after expanded dimension have the same shape. Given a scalar inner product, we get a vector with shape [batch size, units of the second expert hidden layer, units of the second gate hidden layer]. We do reduce-sum operation in the last dimension, which calculates the final expert gate output. The shape is [batch size, units of the second expert hidden layer].

### 2.2. Multitask Learning in Recommendation

In recommendation scenario, the parameters that can be debugged for multitask learning mainly include the following:

1. Label weight: it is similar to the class weight in the deep neural network configuration, to control the sample ratio of each label.
2. Loss weight: setting the weight of loss function for each task. The parameter needs to be adjusted by multiple rounds, and then the optimal combination is selected.
3. Export weight: the weight for predicting score of each task, which can be set higher weight for the better task based on the test result.
4. Task number: setting the number of tasks.
5. Expert number: the number of experts. Each expert is a two-layer fully connected network. The prediction scores are weighted by the output of gating network as the input of the tower network of each task.
6. The number of experts' layers.
7. The number of hidden units.
8. The number of gate network's layers.
9. The number of tower network's layers.

**Figure 2:**
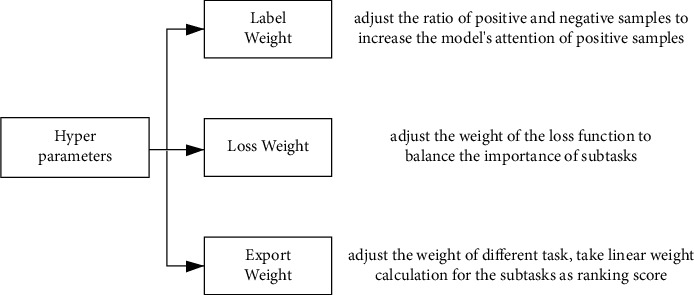
The parameters turning architecture of core multitask learning.

The parameters turning of core neural network is as shown in Figure 2.

**Figure 3:**
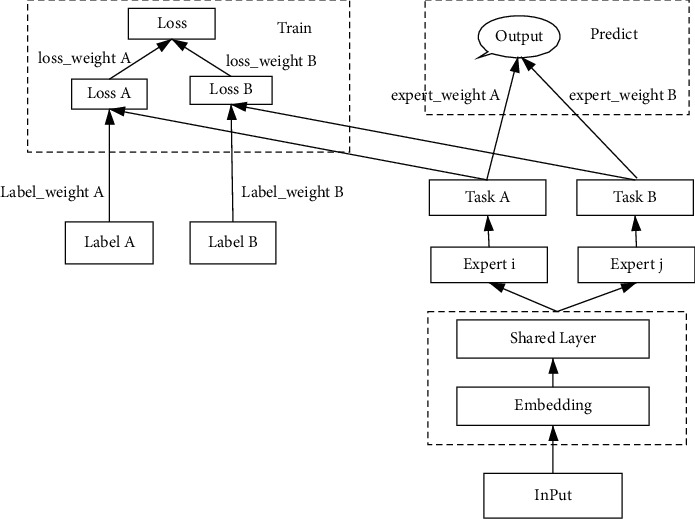
Flowchart of the model training mechanism.

The model training mechanism is as shown in Figure 3.

## 3. The Proposed Scheme

We think there are two parts where MMOE can be improved. The first point is how experts share the parameters with each other, and how to add attention mechanisms effectively. The second point is the design of the loss function, and how to balance the learning of different tasks.

### 3.1. Coarse-Grained Attention Gate Network

In MMOE model, gate network is a linear transformation, which learns parameters from the original features. The expression skills of gate are insufficient. We use the attention mechanism to calculate the model weights, which are updated with the model trained. We improve the calculation of the original gate network, which is from a linear translation to an inner product operator.

**Figure 4:**
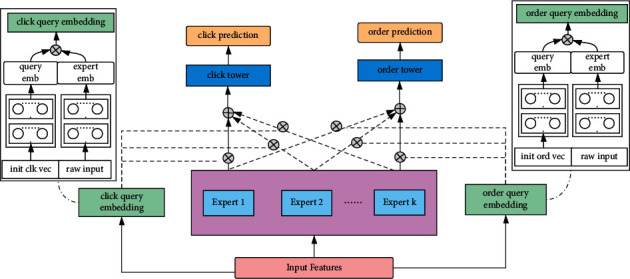
The framework of coarse-grained attention network.

By the guidance of the experts, the model weights are constructed. The design of gate network introduces the prior knowledge of experts. From the view of expert neuron dimension, the output of each neuron is different. Attention is added in the neuron dimension. We add weights in the gate control perspective, and change the gate attention mechanism. We make the improvements on the basis of MMOE, which is shown in Figure 4.

Gate improvement part is as Figure 4 shows. MMOE calculates the weights of different experts by fusing the original feature and gate net output. Inspired by the attention mechanism, each task layer learned a query vector for each expert network. Take inner product between the query vector and the expert network. Then regard the result of inner product as the attention weight of the task's corresponding expert.

The improvement scheme is expressed in the following formula:

(6) $$\begin{array}{c} y_{e}=f_{e}\left(w_{e1}*X*w_{e2}+b_{e}\right), \end{array}$$

where *X* represents the original input, *w*_*e*1_ and *w*_*e*2_ denote the matrix parameters of expert network, *b*_*e*_ is the bias of expert network, and *f*_*e*_(.) represents the transformation function from the original input to the expert vector.

(7) $$\begin{array}{c} y_{g}=\sigma \left(w_{g}*e_{g}+b_{g}\right), \end{array}$$

where *w*_*g*_ is the parameter of the gate network, *e*_*g*_ is the query vector for the initialize gate network, *b*_*g*_ is the bias of the gate network, and *σ* represents the mapping operator.

(8) $$\begin{array}{c} y_{att}=h\left(y_{e}\right)⊙t\left(y_{g}\right), \end{array}$$

where *h*, *t* denote transform function and ⊙ means inner product operation.

Gate-improved attention is more associated with expert matching and more specific to task representation.

#### 3.1.1. The Part of Expert

*Step 1*. Build a neural network for each expert and get the output *y*.
  (9) $$\begin{array}{c} Y=\left[N,F\right]*\left[F,256\right]*\left[256,128\right]=\left[N,128\right]. \end{array}$$
*Step 2*. Build a list of experts output, which stores the result of expert.
*Step 3*. Stack the experts output in the last dimension, and the tensor shape is [*N*, 128,8].

#### 3.1.2. The Part of Gate Network Improvements

 
*Step 1*. Build a neural network for each gate. The gate has one layer with a shape of [1,128], in which 128 is the number of neurons in the last layer of MMOE expert units.
 
*Step 2*. Store the gates output of each task in the dictionary named gates output.
 
*Step 3*. Stack experts output in the second dimension and calculate the expert's result with the tensor shape is being [*N*, 8,128]. We construct expert weight query vector for each task. The query vector is obtained by multiply product operation of gate output [1,128] and expert [*N*, 8,128].
 
*Step 4*. Make elementwise operation on gates and expert output with expanded dimensions, using the broadcast mechanism. We obtain the initial query vector with the shape of [*N*, 8,128], and then aggregate using reduce-sum function in the last dimension. We get attention dot tensor with the shape of [*N*, 8].
 
*Step 5*. By expanding and copying the attention dot tensor, we calculate the expert weights with the shape of [*N*, 128,8]. The shape of weights and the shape of experts are the same.
 
*Step 6*. We add weights for the experts and calculate the final output of [*N*, 128].

Our main improvement is using the expert information to design a query vector for each gate, by the attention mechanism. The fine-grained attention based on the coarse-grained attention makes different weight values in the embedding dimension. The description about fine-grained attention is shown in following part.

### 3.2. Fine-Grained Attention Gate Network

In the dimension of the expert neuron and the dimension of the embedding, we employ attention together. In this way, the gate control network is not only a simple two-layer fully connected network, but also the result of combining the initial gate with the expert by attention mechanism. The model learns the fine-grained query vector for each task.

#### 3.2.1. Expert Network Part

It is the same as the expert network part of MMOE coarse-grained attention.

#### 3.2.2. Attention Gate Network Part

The coarse-grained attention constructs the neural network for each gate with the shape of [1,128]. And then, the gate network and expert with the shape of [*N*, 8,128] make multiply product operation. We design query network for each task with the shape of [*N*, 8,128], in which 128 dimensions are different while 8 dimensions are the same. The fine-grained attention is different in both 128 dimensions and 8 dimensions, which can better adapt the different correlation tasks.

### 3.3. Gradient Boosting Expert Network

In MMOE model, the experts can be regarded as random forest. In order to make different experts interact better, we improve the experts' mode from random forest to gradient boosting decision tree. We construct an expert list named hub-list, which is used to store the output of each expert. When the hub-list is traversed, the information will be appended at the end of the list. If there is no element in the expert hub center, we feed the previous extracted feature into the neural network. If there are elements in the expert hub center, we feed the last layer of the expert hub jointed with the previous extracted feature into the neural network. The idea that the random forest is improved to the gradient boosting tree mainly occurs in the expert part.

#### 3.3.1. Expert Network Improvement Part

We set up an expert output, which is used to store the prediction score of each expert. If it is the first expert, the receiving input is the original feature. If it is a latter expert, the receiving input is the original feature and the prediction value of the former expert. By this way, it is equivalent to increasing the number of feature columns. With the construction of neural network, it has no effect on the final outputs of expert. The shape is [*N*, 128], and after stacking, it is [*N*, 128,8].

#### 3.3.2. Gate Network Part

Like the native MMOE model, we construct a neural network, whose output shape is [*N*, 8]. And then we expand the dimension and turn it into [*N*, 128,8]. By the tensor of this shape, we add weights for the experts. We aggregate and calculate the output with shape of [*N*, 128] finally.

### 3.4. Explicit Self-Attention Expert Interaction

In the paper [21], the method of self-attention is used to interact among different features. Drawing lessons from this idea, we regard the output of different experts as abstract high-level features, and design an interactive network layer.

**Figure 5:**
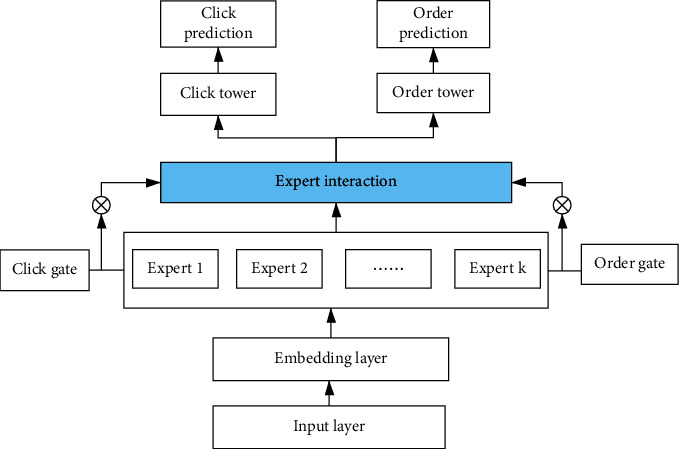
The framework of explicit self-attention expert interaction.

As is shown in Figure 5, on the basis of MMOE, we add an expert interaction layer, using a multi-head attention mechanism. The output after the interaction is used as a high-order feature. We employ an inner product operation between expert output and the high-order feature, and feed the results into the tower network of each task. By automatic interaction, the knowledge could be learned from experts to mine the user interests better.

Specifically, we adopt the key-value attention mechanism to capture combination among different experts. Taking the expert *m* as an example, we define the correlation between expert *m* and expert *k* under a specific attention head *h* as follows:

(10) $$\begin{array}{c} \alpha_{m,k}^{\left(h\right)}=\frac{\exp\left(f^{h}\left(e_{m},e_{k}\right)\right)}{\sum_{l=1}^{M}\exp\left(f^{h}\left(e_{m},e_{l}\right)\right)}, \end{array}$$

where *f*^*h*^(·) is an attention function, *e*_*m*_ denotes the expert *m*, and *e*_*k*_ denotes the expert *k*; in this work, we employ inner product as attention function.

(11) $$\begin{array}{c} f^{h}\left(e_{m},e_{k}\right)=\langle W_{\text{query}}^{\left(h\right)}e_{m},W_{\text{key}}^{\left(h\right)}e_{k}\rangle ,\\ {\tilde{e}}_{m}^{\left(h\right)}=\sum_{k=1}^{M}\alpha_{m,k}^{\left(h\right)}\left(W_{\text{value}}^{\left(h\right)}e_{k}\right), \end{array}$$

where *W*_query_^(*h*)^ and *W*_key_^(*h*)^ are transformation matrices that map the original expert space into a new space. *W*_value_^(*h*)^ is the value space matric, and ${\tilde{e}}_{m}^{\left(h\right)}$ is vector of expert *m* (under head *h*); furthermore, we combine *h* head as expert-output.

Feature-level multi-head self-attention is introduced to feature engineering, and then input to the expert network. The result is worse than the expert-level mode, so we choose the better one.

### 3.5. Deep Interest Sequence Feature Applied into Multitask Learning

**Figure 6:**
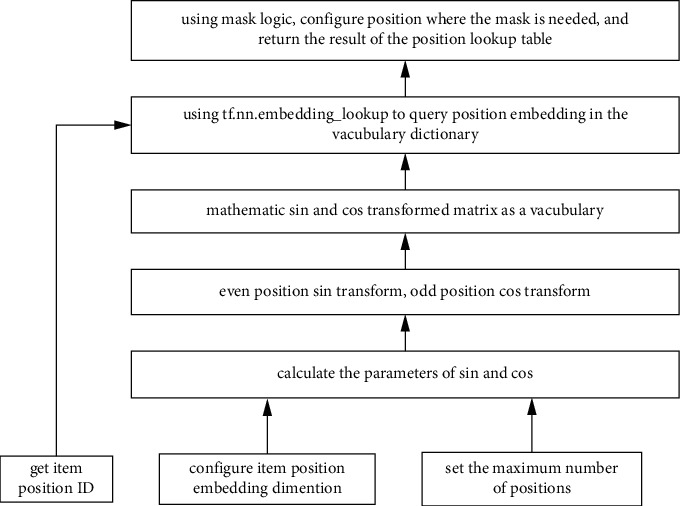
The spatial information embedding method.

The improved MMOE_DIN model introduces the sequence feature to bottom layer. The sequence feature can capture the correlation of user's behavior better. The underlying features are processed by the way of deep interest network. On the basis of user sequence features, we design the embeddings, which represent spatial information and time information. The spatial information embedding method is as shown in Figure 6.

**Figure 7:**
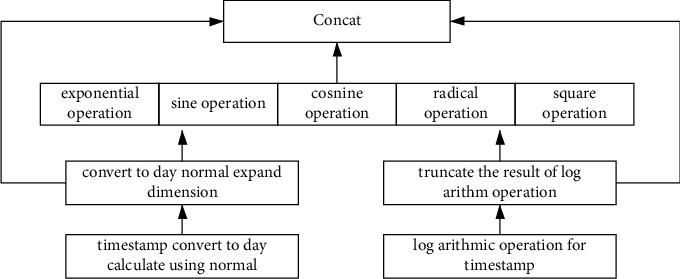
The time-embedding information method.

The time-embedding information method is as shown in Figure 7.

We normalize the timestamp as days, and make some mathematic operations. The mathematic operations include exponential function operation, sine function operation, cosine function operation, root operation, square operation, and logarithmic operation. And then, we concatenate them into a large embedding vector.

### 3.6. Improve Loss Function with Multitask Learning

**Figure 8:**
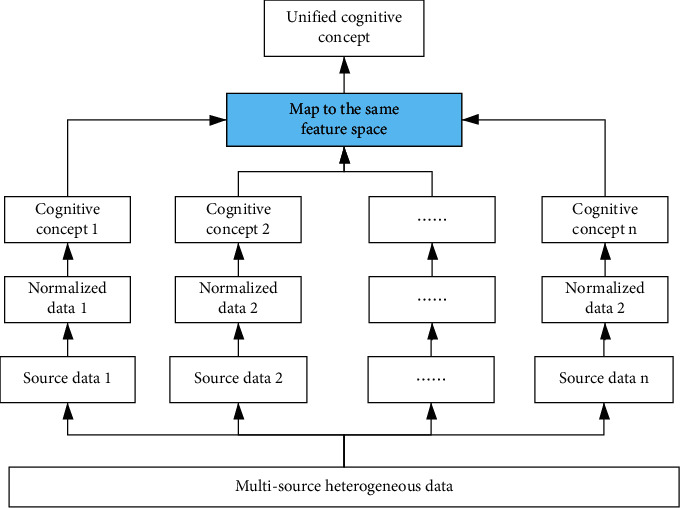
The framework of cognitive intelligence with multitask learning.

Recently, artificial intelligence is gradually developing from the perceptual intelligence to cognitive intelligence. Deep learning is the mainstream technology in the recommendation system rank stage. More and more scholars [22, 23] try to introduce cognitive intelligence into recommendation. Recommendation system has multiple scenarios, and the data is heterogeneous. Traditional multitask learning joint training requires data feature to be aligned. Combining heterogeneous data from multiple scenarios to train model, we propose a feature space mapping operator. The above operator can project the heterogeneous data into the same feature space via processing multiple network layers. From the perspective of cognitive intelligence, it is easier for multiple experts to share collective wisdom in the same feature space. The data cognition fusion scheme is as shown in Figure 8. For the cognitive learning of multitask shared parameters, we design a custom loss function. In the learning process, the features extracted from the current data source are regarded as real data, and its label is set as real label. The features extracted from the other data sources are regarded as fake data, and the corresponding labels are set as fake label. In this way, in multitask learning, with the multisource feature iteration training, the discriminator is difficult to distinguish the shared data sources, so as to achieve the shared cognitive effect.

The multitask learning model makes feature space mapping for the data from different sources so that the multisource data are in the same feature space. We construct the following cognitive loss function, where *c*_*i*_^*k*^ is real or fake label, and add it to basic loss function.

(12) $$\begin{array}{c} L_{\text{improve}}=\sum_{k=1}^{K}\sum_{i=1}^{N_{k}}c_{i}^{k}\log\left(D\left(S_{k}\left(i\right)\right)\right). \end{array}$$

## 4. Experiment

In this section, we evaluate the performance of our proposed novel model on the public Ali-CCP data. Experimental comparison shows the effectiveness of our model, which outperforms the state-of-art methods for multitask learning.

### 4.1. Datasets

The public dataset Ali-CCP containing 42 million train samples and 43 million test samples, which extracted from Taobao's Recommender System. The train dataset storage is 10G, and the test dataset storage is 8G. CTR and CVR are two tasks modeling actions of click and purchase in the dataset. The dataset contains labels section and features section. The labels consist of click label and conversion label. The features consist of feature field id, feature id, and feature value. Features include user features, item features, combination features, and context features. The data detail instruction is in the page below (https://tianchi.aliyun.com/dataset/dataDetail?dataId=408&userId=1). We randomly select 10% of the train dataset as the validation dataset to test the evaluate index of all models.

### 4.2. Baseline Models

We compare our proposed model with the following baseline and mainstream models:

 
*MLP* [24]. We use the Multi-Layer Perceptron structure as our baseline, which is a single-task model.
 
*Shared Bottom* [25]. The model with Expert-Bottom pattern shares several low-level network layers for all the tasks, and each task has its own tower.
 
*ESMM* [4, 26]. The model with Probability-Transformer pattern is used to predict the post-click conversion rate, which can relieve the sample selection bias problem via training on the entire space.
 
*OMOE* [2]. The model with Expert-Bottom pattern integrates experts by sharing one gate among all tasks.
 
*MMOE* [2]. The model with Expert-Bottom pattern integrates experts by multiple gates among all tasks.
 
*CGC* [7]. The model with Expert-Bottom pattern separates task-shared experts and task-specific experts, which is designed to solve the multitask negative transfer problem.
 
*PLE* [7]. The Progressive Layered Extraction (PLE) with Expert-Bottom pattern, and is made up by multilayer CGC.

Using Ali-CCP dataset, we adopt a two-layer MLP network with DICE activation and hidden layers for each task in both MTL models. Hyperparameters turned are shown in Table 1.

### 4.3. Experiment Setting

Hyperparameter Study

In order to study the effectiveness of hyperparameters, we try the random search, grid search, and anneal methods.

**Figure 9:**
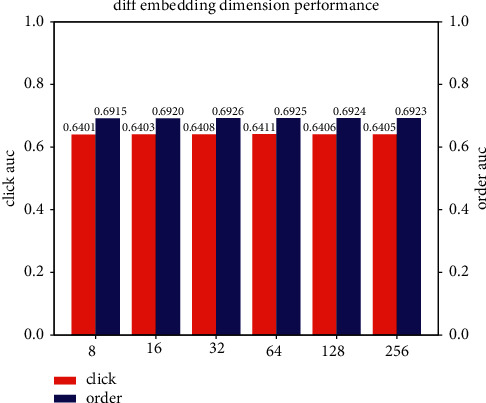
The AUC of different embedding dimension.

**Figure 10:**
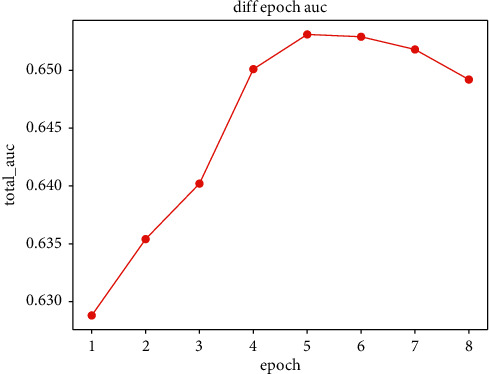
The total AUC of different epochs.

**Figure 11:**
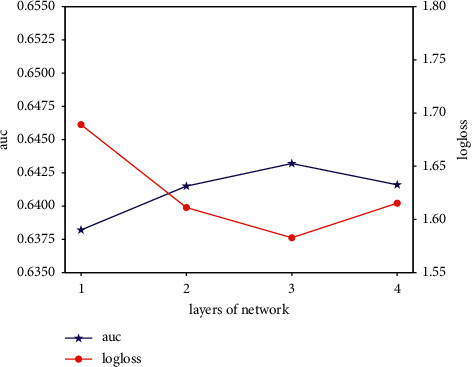
The AUC and log-loss of different network layers.

1. Considering the category embedding dimension, we do experiment by varying embedding dimension [8, 16, 32, 64, 128, 256, 512, 1024], and the results are shown in Figure 9. We can see that the effects of the model are slightly affected by the embedding dimension. The embedding dimension is related to the model complexity and volume.
2. Smaller embedding dimension leads to fitting the data distribution insufficiently, while larger embedding dimension increases the model complexity; proper embedding dimension will produce the best effect. Making a trade-off between the fitting ability and complexity, we finally select embedding dimension = 32 in all the experiments.
3. We study the impact of export weight; there is seesaw phenomenon among two different tasks. However, the export weight brings improvement overall the performance. We finally set the export weight of click task as 0.8 and order task as 0.2.
4. We study the impact of epoch number in the dataset and report the AUC performance on the entire test dataset as shown in Figure 10. We finally set the epoch number as 5 in all the experiments.
5. We study the number of layers in our proposed model; the effectiveness of AUC and log-loss is as follows. As the number of neural network layers increases, the AUC first increases then decreases and the log-loss is opposite trend. Therefore, we finally choose 3 layers in all experiments, which is as shown in Figure 11.

### 4.4. Experiment Results

Compared with the baseline MMOE, ESMM, and CGC, we demonstrate the effectiveness of our approach on Ali-CCP public dataset. We show that the proposed method improves the accuracy of multitask models. Offline evaluator of our model brings significant improvement. In order to obtain accurate prediction results, we repeat experiments 5 times for each model, among which the best offline effect is shown in Table 2.

To evaluate the effectiveness of our proposed model, we adopt four widely used metrics in experiments, i.e., AUC, Log-loss, CLICK@2, and ORDER@2.

AUC: area under curve, which reflects the ranking ability. The score ranges from 0 to 1, and the higher the better. The AUC formula is as follows:

(13) $$\begin{array}{c} \text{AUC}=\frac{1}{\left|D_{+}\right|\left|D_{-}\right|}\sum_{x^{+}\in D_{+}}\sum_{x^{-}\in D_{-}}I\left(f\left(x^{+}\right)>f\left(x^{-}\right)\right), \end{array}$$

where *D*_+_ and *D*_−_ denote the set of positive and negative samples, |*D*_+_| and |*D*_−_| mean the number of samples in *D*_+_ and *D*_−_, *f*(.) is the prediction function, and *I*(·) is the indicator function.

*Log-Loss*. In multitask learning, a common equation of joint log-loss is the weighted sum of the individual task log-loss.

(14) $$\begin{array}{c} L\left(\theta_{1},\ldots ,\theta_{K}\right)=\sum_{k=1}^{K}w_{k}L_{k}\left(\theta_{k}\right), \end{array}$$

where *K* is the number of tasks, *L*_*k*_(·) is the loss function, *w*_*k*_ is the loss weight, and *θ*_*k*_ is the task parameters.

(15) $$\begin{array}{c} L_{k}\left(\theta_{k}\right)=y_{k}\times \left(-\log\left(\text{sigmoid}\left({\hat{y}}_{k}\right)\right)\right)+\left(1-y_{k}\right)\times \left(-\log\left(1-\text{sigmoid}\left({\hat{y}}_{k}\right)\right)\right), \end{array}$$

where *y*_*k*_ denotes the real label, ${\hat{y}}_{k}$ denotes the predict value, and sigmoid is the activate function.

*CLICK@2*. It is the probability of actual click number in the prediction top *N* score.

(16) $$\begin{array}{c} \text{CLKICK}@2=\frac{{\left|top\left({\hat{y}}_{n}\right)\right|}_{n}}{N}. \end{array}$$

*ORDER@2*. It is the probability of actually buy number in the prediction top *N* score.

(17) $$\begin{array}{c} \text{ORDER}@2=\frac{{\left|\text{top}\left({\hat{y}}_{n}\right)\right|}_{n}}{N}, \end{array}$$

where *n* denotes the number of real click/buy sample in the top *N* score, and *N*equals 2 in our paper.

In order to reduce the accidental error of the experiment, we repeat the training process of each improved model for 5 times.  Table 3 shows the average increase of 5 times for each model.

As mentioned above, in order to increase the credibility of the experiment, we repeated the training process 5 times for each model.

Custom evaluation indicators: In order to compare model effects more fairly, we evaluate models from multiple perspectives. Besides AUC, we customize two categories of offline evaluation indicators: CLK@*N* and ORD@*N*.

CLICK@*N*: In the top *N* commodities recommended by the model, the proportion of the number of commodities which the users click.

ORDER@*N*: In the top *N* commodities recommended by the model, the proportion of the number of commodities which the users purchase.

In order to reduce the accidental error of the experiment, we repeated the training process of each improved model for 5 times.  Table 4 shows the average of 5 custom evaluation for each model.

From all the above tables, we can see that our methods bring positive improvements.

### 4.5. Ablation Study

**Figure 12:**
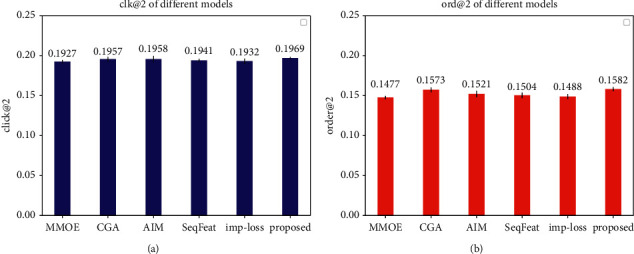
Click@2 and order@2 of different models.

From Tables 2–4, comparing to base MMOE, we can see that every proposed point has improvement. Sequence feature can bring +3.65% in AUC due to the feature engineering improvement. Coarse-grained attention can bring +3.41% in AUC, and fine-grained attention can bring +2.11% in AUC. Coarse-grained and fine-grained are two patterns of attention methods. We choose coarse-grained considering the fine-grained attention may lead to overfitting. Boosting expert mode and auto interact layer mode are all used to describe the expert interaction, and we select the auto interact layer because it performs better. Furthermore, we improve the loss function to better support multisource datasets feeding, and the model structure is more generic. Finally, we integrate the above four methods, and the prediction effect is significantly improved. click@2 and order@2 of each model are shown as in Figure 12. The experiment is repeated 5 times and the error fluctuation is small. It can be seen that our new integrating model has the best effect.

## 5. Conclusions

In this paper, we propose five improvement methods about the multitask learning, which focus on the expert interaction and gate attention mechanism. In the public data set, there is a significant improvement comparing with the MMOE model. We optimize the gate network, which relies on introducing the coarse-grained and fine-grained attention mechanism. By a linear transformation, the gate network of native MMOE pays more attention to the expert using the original input, so the expression ability is insufficient. We calculate the weights of the gate using the attention mechanism. We upgrade the calculation of the gate network, which is from a linear transformation to multiple matrix inner product operations. We introduce the gradient boosting tree in the MMOE experts, which improve both the knowledge representation and the efficiency of mutual communication reasoning. Multihead attention is applied on the expert feature extraction layer, which can represent high-order features better. In addition, we fuse sequence DIN and MMOE, which make the multitask learning consider the relevance of features.

In further work, we will introduce cognitive intelligence in multitask learning more. The cognitive intelligence can give full play to the wisdom of experts. Expert system based on frames and expert system based on models are regarded as different experts in the multitask learning algorithm. We will build a broader recommendation system, which use multi-experts and multitask to work collaboratively.

## Figures and Tables

**Table 1:** Hyperparameter settings.

Hyperparameter	Value
Label weight	Positive sample, negative sample = 1 : 1
Loss weight	Click task, order task = 1 : 0.02
Export weight	Click task, order task = 0.8 : 0.2
Task number	2
Expert number	8
Hidden unit	256 128 64
Learning rate	0.001
Batch size	1000
Epoch	5

**Table 2:** The performance of different models.

Models	Click AUC best	Order AUC best	Loss
MMOE (base1)	0.6209	0.6645	1.6027
ESMM (base2)	0.6203	0.6712	1.6105
CGC (base3)	0.6311	0.6708	1.6112
Coarse-grained attention	0.6395	**0.6957**	1.5843
Fine-grained attention	0.6339	0.6884	**1.5827**
Expert with boost mode	0.6409	0.6804	1.7268
Add auto interact layer	**0.6432**	0.6824	1.6891
Sequence MMOE	0.6413	0.6870	1.6152
Improve loss function	0.6407	0.6924	1.5997
Coarse-grained attention + auto interact layer + sequence feature + improve loss function	**0.6513**	**0.6966**	**1.5784**

**Table 3:** The improvements of different models.

Models	Clk AUC improve	Ord AUC improve
MMOE (base)	Baseline 1	Baseline 1
ESMM (base)	Baseline 2	Baseline 2
CGC (base)	Baseline3	Baseline 3
Coarse-grained attention	+2.46%	**+4.00%**
Fine-grained attention	+1.57%	+2.91%
Expert with boost mode	+2.69%	+1.74%
Add auto interact layer	**+3.06%**	+2.02%
Sequence MMOE	+2.75%	+2.71%
Improve loss function	+2.65%	+3.51%
Coarse-grained attention + auto interact layer + sequence feature + improve loss function	**+4.35%**	**+4.14%**

**Table 4:** The custom evaluation of different models.

Models	CLICK@2	ORDER@2
MMOE	0.1927	0.1477
ESMM	0.1925	0.1490
CGC	0.1931	0.1487
Coarse-grained attention	0.1957	**0.1573**
Fine-grained attention	0.1953	0.1463
Expert with boost mode	0.1936	0.1478
Add auto interact layer	**0.1958**	0.1521
Sequence MMOE	0.1941	0.1504
Improve loss function	0.1932	0.1488
Coarse-grained attention + Add auto interact layer + Sequence MMOE + improve loss function	**0.1969**	**0.1582**

## Data Availability

The Ali-CCP public dataset has been used in the experiments. Ali-CCP dataset is a public dataset containing 84 million samples extracted from Taobao's Recommender System. CTR and CVR (Conversion Rate) are two tasks modeling actions of click and purchase in the dataset. The dataset url is https://tianchi.aliyun.com/dataset/dataDetail?dataId=408.
